# Factors influencing time from presentation to treatment of colorectal and breast cancer in urban and rural areas

**DOI:** 10.1038/sj.bjc.6601753

**Published:** 2004-03-30

**Authors:** R Robertson, N C Campbell, S Smith, P T Donnan, F Sullivan, R Duffy, L D Ritchie, D Millar, J Cassidy, A Munro

**Affiliations:** 1 Department of General Practice and Primary Care, University of Aberdeen, Foresterhill Health Centre, Westburn Road, AB25 2AY Aberdeen, UK; 2 Tayside Centre for General Practice, Kirsty Semple Way, DD2 4AD Dundee, UK; 3 The Beatson Oncology Centre, Dumbarton Road, G11 6NT Glasgow, UK; 4 Raigmore Hospital, Old Perth Road, IV2 3UJ Inverness, UK

**Keywords:** breast cancer, colorectal cancer, provider delay, cohort study, primary care

## Abstract

Stage at diagnosis and survival from cancer vary according to where people live, suggesting some may have delays in diagnosis. The aim of this study was to determine if time from presentation to treatment was longer for colorectal and breast cancer patients living further from cancer centres, and identify other important factors in delay. Data were collected on 1097 patients with breast and 1223 with colorectal cancer in north and northeast Scotland. Women with breast cancer who lived further from cancer centres were treated more quickly than those living closer to cancer centres (*P*=0.011). Multilevel modelling found that this was largely due to them receiving earlier treatment at hospitals other than cancer centres. Breast lump, change in skin contour, lymphadenopathy, more symptoms and signs, and increasing age predicted faster treatment. Screen detected cancers and private referrals were treated more quickly. For colorectal cancer, time to treatment was similar for people in rural and urban areas. Quicker treatment was associated with palpable rectal or abdominal masses, tenesmus, abdominal pain, frequent GP consultations, age between 50 and 74 years, tumours of the transverse colon, and iron medication at presentation. Delay was associated with past anxiety or depression. There was variation between general practices and treatment appeared quicker at practices with more female general practitioners.

For all cancers, stage at diagnosis is important for survival. For example, 5-year survival from colorectal cancer varies from 87% for tumours limited to the bowel wall (Dukes A) to 15% for advanced tumours in whom only palliative resection is undertaken (McArdle and Hole, 2002). There is concern that the relatively poor survival rates from cancer reported in the United Kingdom may be caused by relatively late stage disease at diagnosis and that this may reflect delays before treatment (Coebergh *et al*, 1998; Gatta *et al*, 2000). Within the United Kingdom, chances of both early diagnosis of and survival from cancer vary according to where people live. In Scotland, for several common cancers, increasing distance from cities with cancer centres is associated with more advanced disease at diagnosis and poorer early survival (Campbell *et al*, 2000, 2001). This accords with studies in Europe and the United States (Liff *et al*, 1991; Launoy *et al*, 1992).

The principle that all patients wherever they live should have access to a uniformly high quality of care to ensure the best possible survival rates was laid down by the Expert Advisory Group on Cancer in England and Wales and endorsed in Scotland (EAGC, 1995; SCCAC, 1996). Recognising the importance of stage at diagnosis, the cancer plans have set targets in an attempt to speed up time to treatment (NHS, 2000). Little is known, however, about factors that influence delay in diagnosis, nor how they affect delay at the various levels where it can occur. Delays in diagnosis can occur before presentation (patient delay) or between initial presentation and treatment (provider delay). This study aimed to determine for patients with breast and colorectal cancer if the time between first presentation and specialist treatment was longer for patients who lived further from cancer centres than for patients who lived nearby, and to discover which other patient, general practice and secondary care characteristics were associated with time to treatment.

## METHODS

People resident in the Grampian, Tayside or Highland Health Board areas of Scotland who were diagnosed with a first primary tumour of colorectal or breast cancer (female) between 1 January 1997 and 31 December 1998 were identified from the Scottish Cancer Registry (Brewster *et al*, 1997). Their names, dates of birth and death, sex, postcodes and health boards of residence at time of diagnosis, cancer sites, practices registered with at time of diagnosis, incidence dates and histories of previous cancers were obtained from the registry. Information on dates of death had been provided by the General Register Office (Scotland) and linked with the cancer registration record by record linkage at the Information and Statistics Division of the NHS in Scotland (Kendrick and Clarke, 1993). Cases that were duplicates, or could not be matched to 1991 census output areas, or were not allocated to practices in the study areas were excluded.

Permission was requested from all general practitioners in the study area with eligible patients to review medical records. Patients who had moved from practices were excluded from the study. Records of deceased patients were requested from the Practitioner Services Division of the NHS in Scotland. Data collected from medical records between May 2001 and May 2002 included presenting symptoms and clinical signs, dates and places of consultations, referral route and destination, date of first treatment (surgery, radiotherapy, chemotherapy or hormone therapy) and previous history (cancer, breast/bowel comorbidity, breast/bowel investigations with benign results, anxiety, depression, iron medication and smoking status).

A ‘2-year rule’ was used to reduce the likelihood of recording symptoms unrelated to the diagnosis of cancer. Working back in time from the date the cancer was diagnosed, the date was recorded of the first entry in the GP consultation notes of a relevant sign or symptom that appeared more than 2 years after any previous such sign or symptom. The list of eligible symptoms and signs was collated mainly from SIGN guidelines (SIGN, 1998, 2003).

Aggregated data from the 1991 Census were used to calculate distances to cancer centres and deprivation categories (each containing a fifth of the population of Grampian, Tayside and Highland) at the smallest level possible, the output area (Campbell *et al*, 2000, 2001). These were matched to postcodes of residence. Information on practice list sizes, and number and sexes of partners in a practice was obtained from an Information and Statistics Division database of Scottish general practitioners. Finally, information about the type of out of hours provision and waiting times for patients to get an appointment with the doctor of their choice was obtained from staff at practices visited.

Data were managed using Microsoft Access. Descriptive analyses were conducted using SPSS for Windows release 11 and multilevel modelling using MlwiN (Leyland and Goldstein, 2001). Data for breast and colorectal cancer were analysed separately. Our outcome was time from first presentation with suspicious symptoms or signs to first treatment. Our main independent variable (and the subject of our *a priori* hypothesis) was distance to a cancer centre, which was measured as the straight-line distance from the grid reference of output area of residence centroid (from Census data) to the nearest cancer centre (from the postcode directory) (Campbell *et al*, 2000, 2001). We also investigated relationships with time to treatment for multiple symptoms and variables – these analyses should be considered exploratory with statistical significance indicated for *P*<0.002.

In initial univariate analyses, times between presentation and treatment were highly skewed and so were log transformed to achieve a Normal distribution and have been converted back to geometric means and 95% confidence intervals for presentation. This initial analysis has limitations, however, because it excludes patients who were not treated, and does not take account of other significant factors or the structure of the data in four levels – patient, general practice, hospital of first referral and health board. In further analysis, multilevel modelling was employed using binary outcomes for delay (>30 days for breast cancer and >90 days for colorectal cancer) to prevent any bias that might have occurred had those who were not treated been excluded. These cut points were defined *a priori* as close to median expected delays and so results are presented in terms of odds ratios for delay along with 95% confidence intervals. All variables were considered in the multiple logistic regression models as all were considered to be clinically important. Initially, variables with *P*<0.10 (univariately) were entered together and all other variables were added individually and assessed at the conventional 5% level. Finally, a stepwise procedure was performed and compared with the final model for differences. All final models were assessed for extra-binomial variation.

Our proposed sample size was 2000 (1000 each with breast and colorectal cancer). Allowing for an attrition rate of 10–20%, this was calculated to have 80% power to detect an absolute difference of 10% in the proportions of cases waiting more than 30 days (breast cancer) or 90 days (colorectal cancer) for specialist treatment at the 5% significance level in two group comparisons. Ethical approval for the study was sought and obtained from Grampian, Tayside and Highland Research Ethics Committees.

## RESULTS

**Figure 1:**
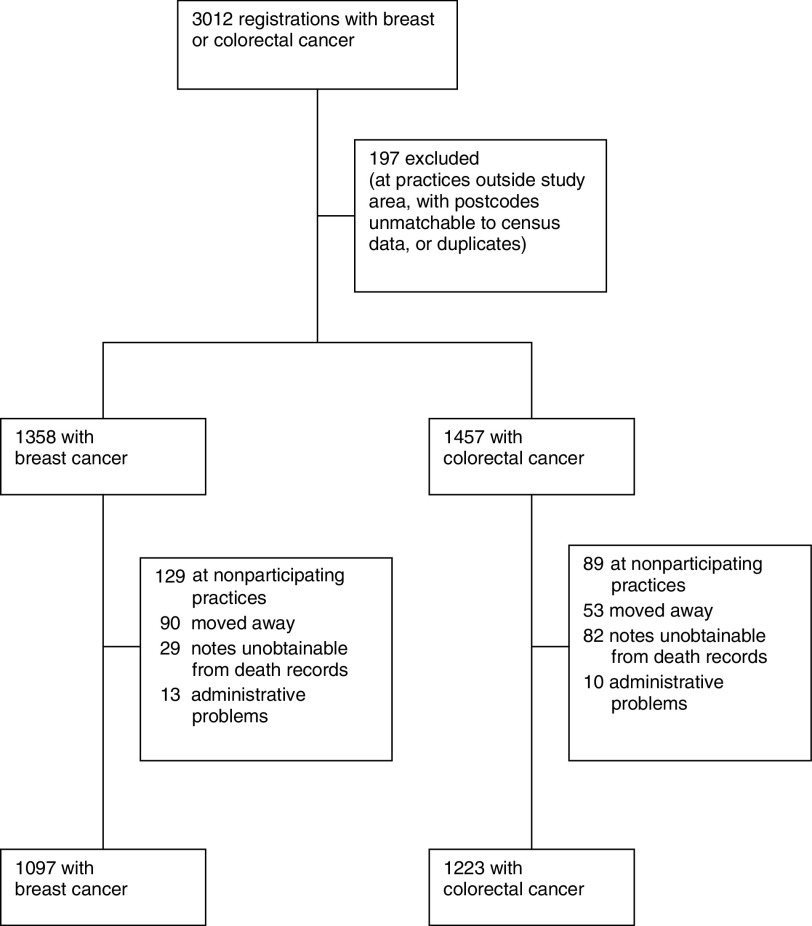
Study profile.

There were 1358 eligible people with breast cancer and 1457 with colorectal cancer (Figure 1

). Of 217 general practices with live patients in the study area, 196 (90%) responded and 187 (86%) agreed to case notes review. The 218 (8%) live patients from practices not agreeing were omitted from the study, along with 143 (5%) who were no longer on their original general practice list, 111 (4%) deceased patients whose notes were unobtainable, and 23 (<1%) for various administrative reasons (principally case notes unobtainable at practice visits). General Practitioner medical records were reviewed for 1097 (80.8%) breast cancer cases of which 1069 had a time from presentation to treatment and 1223 (83.9%) colorectal cancer cases, of which 1071 had a time from presentation to treatment. The main reason for no time to treatment was death before treatment. There were few important differences in available characteristics of patients whose notes were and were not reviewed, but those not reviewed tended to have been younger (mean age 63 *vs* 67 years) and alive at the start of the study (79 *vs* 61%).

### Breast cancer

The geometric mean for time from presentation to treatment was 42 days (95% confidence interval 40–45). On univariate analysis, patients living further from cancer centres tended to be treated more quickly than those living nearby – the fastest time of 36 days (30–42) was for people between 38 and 57 km away (Table 1

**Table 1:** Time to treatment of breast and colorectal cancer according to geographic, organisational and basic demographic variables

	**Breast cancer (*N*=1069)**			**Colorectal cancer (*N*=1071)**		
	***N* (%)**	**Days – geometric mean (95% CI)**	***P*-value^a^**	***N* (%)**	**Days – geometric mean (95% CI)**	***P*-value^a^**
*Distance from cancer centre*			0.017			0.361
0–5 km	297 (28)	43 (38–47)	(0.011)	317 (30)	131 (112–154)	(0.248)
6–13	124 (12)	50 (42–60)		123 (12)	170 (135–214)	
14–23	135 (13)	51 (43–60)		132 (12)	144 (113–183)	
24–37	161 (15)	41 (34–49)		125 (12)	157 (121–204)	
38–57	193 (18)	36 (30–42)		193 (18)	138 (110–173)	
58+	159 (15)	40 (34–46)		181 (17)	119 (94–151)	
						
*Age group (years)*			<0.001			0.038
<50	227 (21)	56 (49–65)	(<0.001)	53 (5)	182 (129–258)	(0.847)
50–64	391 (37)	45 (42–49)		220 (20)	120 (100–145)	
65–74	207 (19)	41 (35–47)		570 (53)	132 (116–150)	
75+	244 (23)	29 (26–34)		228 (21)	169 (139–205)	
						
*Sex*						<0.001
Male				590 (55)	117 (104–132)	
Female				481 (45)	170 (149–194)	
						
*Health board*			0.016			NS
A	227 (21)	42 (37–48)				
B	381 (36)	47 (42–53)				
C	461 (43)	39 (36–42)				
						
*Place of presentation*			<0.001			<0.001
GP	815 (76)	45 (42–49)		1026 (96)	145 (132–159)	
Hospital	56 (5)	27 (18–38)		43 (4)	50 (29–84)	
Mammogram	197 (18)	36 (33–39)				
						
*Hospital referred to*			<0.001			0.001
Cancer centre	808 (76)	45 (42–48)		577 (54)	126 (111–142)	
General	154 (14)	34 (28–41)		277 (26)	133 (110–161)	
Community	77 (7)	49 (35–68)		153 (14)	213 (176–258)	
Private	22 (2)	22 (15–33)		35 (3)	105 (74–151)	
						
*Specialty*			<0.001			<0.001
Medical	21 (2)	20 (9–44)		266 (25)	215 (185–249)	
Surgical	379 (36)	39 (34–44)		787 (74)	121 (109–135)	
Breast clinic	650 (61)	46 (43–49)		14 (1)		
Other					97 (29–330)	
						
*General practice*			NS			
Single handed				59 (6)	208 (151–288)	0.076
2–4 partners				430 (41)	133 (115–153)	(0.172)
5–6 partners				396 (37)	146 (126–170)	
7+ partners				173 (16)	120 (97–148)	

a ANOVA association or linear trend for ordered categories in parentheses. There were no differences (*P*>0.10) for categories of deprivation or other general practice factors (list size, number of female GPs, type of out of hours provision, or waiting time to see GP of choice).

). Older patients were treated more quickly and several organisational variables appeared to be important, but deprivation was not a significant factor. Time to treatment varied significantly according to presenting symptoms – it was fastest for those with bone pain or ulcerating or fungating lesions and slowest for those with bloody nipple discharge (Table 2

**Table 2:** Time from presentation to treatment for presenting breast cancer symptoms and other clinical variables

		**Days from presentation to treatment-geometric mean (95% CI)**		
***N*=1069**	**Number with symptom (%)**	**With symptom**	**Without symptom**	***P*-value**
Breast lump	761 (71)	40 (37–43)	49 (44–55)	0.002
Breast pain	106 (10)	69 (54–88)	40 (38–42)	<0.001
Nipple discharge – blood	9 (1)	168 (50–567)	42 (39–44)	<0.001
Lymphadenopathy (axillary/supraclavicular)	58 (5)	32 (24–41)	43 (40–46)	0.026
Change in skin contour	134 (13)	26 (21–31)	45 (42–48)	<0.001
Ulcerating or fungating lesion	26 (2)	17 (9–30)	43 (41–46)	<0.001
Lump tethered to skin or underlying tissue	50 (5)	31 (23–42)	43 (40–46)	0.033
Bone pain	7 (1)	15 (6–37)	42 (40–45)	0.008
Skin discolouration	12 (1)	25 (13–48)	42 (40–45)	0.080
Fullness/lumpiness/asymmetry	9 (1)	75 (32–176)	42 (39–45)	0.092
				
*Number of symptoms at presentation*				<0.001
0	225 (21)	36 (32–40)		
1	494 (46)	53 (48–58)		
2	248 (23)	37 (33–42)		
3 or more	102 (10)	27 (22–34)		
			
Benign investigations in previous 5 years	292 (27)	49 (45–54)	40 (37–43)	0.002
Past history of benign breast disease	220 (21)	55 (46–64)	39 (37–42)	<0.001

No differences (*P*>0.10) for nonblood stained nipple discharge, nipple distortion, nipple eczema, breast tenderness, discomfort or itch, breast inflammation, lymphoedema, or past history of anxiety or depression.

). Increasing numbers of symptoms at presentation predicted faster treatment, but asymptomatic patients (predominantly detected by screening) were also treated quickly. Treatment was slower for those with previous benign investigations or benign breast disease.

When variables were assessed in a multilevel model, variation was found at individual and hospital of first referral levels, but not general practice or health board (Table 3

**Table 3:** Results of multilevel modelling on treatment within 30 days (yes/no) with variation at patient, hospital of referral and health board level for people with breast cancer

	**Odds ratio (95% confidence intervals)^a^**	
	**All referrals^b^ *N*=1097**	**Referred by GP *N*=822**
Individual variables		
Age at incidence (+10 years)	1.16 (1.05–1.28)	1.12 (0.99–1.26)
*No. of symptoms*		
0 (i.e. screen detected)	11.92 (5.31–26.72)	
1	1	1
2	1.43 (0.95–2.16)	1.62 (1.05–2.51)
3+	1.97 (1.07–3.64)	2.39 (1.26–4.52)
		
Breast lump (yes *vs* no)	3.16 (1.48–6.71)	5.63 (2.20–14.42)
Change in skin contour (yes *vs* no)	1.71 (1.02–2.85)	1.75 (1.03–2.97)
Lymphadenopathy (yes *vs* no)	2.05 (1.06–3.99)	2.26 (1.13–4.53)
		
*Hospital variables*		
Hospital type		
Cancer centre	1	1
General	4.18 (2.15–8.13)	5.09 (2.64–9.79)
Community	2.86 (1.45–5.66)	3.63 (1.81–7.28)
Private	13.38 (4.34–41.22)	15.63 (5.09–47.95)
		
	Variation (standard error) at:	
	Practice level 0.000 (0.000)	Practice level 0.000 (0.000)
	Hospital level 0.129 (0.096)	Hospital level 0.104 (0.094)
	Health board level effectively zero	Health board level effectively zero

a Odds ratios higher than unity mean higher odds of being treated within 30 days. The values of odds ratios (and their differences from unity) provide an indication of effect sizes. The magnitude of odds ratios should be interpreted along with the unadjusted means in the previous tables.

b Includes GP referrals, patients with cancers detected by breast screening and others.

). The main factors associated with odds of treatment before 30 days were clinical, but the number of symptoms that predicted faster treatment condensed to three – breast lump, change in skin contour and regional lymphadenopathy. Again, patients with no symptoms (detected by screening) were treated quickest, but excluding them, treatment was quicker for patients with more symptoms. Only one organisational variable remained significant: treatment was faster for patients referred to noncancer centre hospitals (other general hospitals and community hospital outreach clinics) than for those referred to cancer centres and fastest at private hospitals.

### Colorectal cancer

For colorectal cancer the geometric mean for time from presentation to treatment was 138 days (127–152). On univariate analysis, there were no differences in times from first presentation to treatment for patients living different distances from cancer centres (Table 1). Age was important, with fastest treatment for those in the 50–64 year age group and men were treated more quickly than women. Again, several organisational variables appeared to be important, but health board of residence and deprivation category were not. With regard to symptoms, treatment was fastest for those with abdominal guarding, and slowest for those without abdominal pain (Table 4

**Table 4:** Time from presentation to treatment for presenting colorectal cancer symptoms and other clinical variables

		**Days from presentation to treatment-geometric mean (95% CI)**		
***N*=1071**	**Number with symptom (%)**	**With**	**Without**	***P*-value**
Abdominal pain	318 (30)	102 (83–125)	158 (144–173)	<0.001
Palpable abdominal mass	40 (4)	32 (22–48)	147 (134–160)	<0.001
Rectal bleeding	286 (27)	105 (92–120)	153 (137–171)	<0.001
Rectal mass	31 (3)	46 (35–59)	143 (131–157)	<0.001
Guarding	6 (1)	22 (0.9–540)	140 (128–153)	0.003
Nausea	36 (3)	77 (39–153)	141 (129–155)	0.017
Rectal mucus discharge	47 (4)	85 (60–122)	142 (129–155)	0.023
Tenesmus	29 (3)	76 (49–116)	141 (129–154)	0.028
Increased frequency of bowel movements	67 (6)	99 (72–135)	142 (129–156)	0.057
Abdominal distension	60 (6)	98 (57–170)	141 (129–155)	0.066
Vomiting	83 (8)	105 (65–170)	142 (130–155)	0.083
				
*Number of symptoms at presentation*				<0.001
0	26 (2)	57 (27–119)		
1	459 (43)	208 (184–235)		
2	332 (31)	133 (114–156)		
3	150 (14)	104 (83–131)		
4 or more	104 (10)	49 (36–68)		
				
History of anxiety or depression	143 (13)	210 (167–264)	130 (118–143)	<0.001
Past history of benign bowel disease	168 (16)	169 (133–215)	133 (121–147)	0.059
				
*GP consultations/month*				<0.001
0–0.5	248 (23)	620 (571–673)		
0.5–2.5	247 (23)	182 (164–202)		
2.5–10	222 (21)	92 (82–105)		
>10	283 (26)	49 (40–59)		
				
*Tumour site*				0.072
Right side colon to hepatic flexure	263 (25)	163 (136–197)		
Transverse colon to splenic flexure	105 (10)	102 (73–141)		
Descending and sigmoid colon	286 (27)	140 (116–170)		
Rectosigmoid and rectum	379 (35)	136 (120–154)		
Unspecified or overlapping	38 (4)	111 (66–188)		

No differences (*P*>0.10) for rectal or anal discomfort, melaena, breathlessness, pallor, tiredness, anaemia, weight loss, anorexia, unspecified changes in bowel habit, urgency, diarrhoea, constipation, nonspecific anal symptoms, faecal incontinence or discharge, iron medication at time of presentation, or previous benign colorectal investigations.

). Increasing numbers of symptoms and frequency of GP consultations predicted faster treatment but it was slower for those with a history of anxiety, depression or benign bowel disease. Tumours of the transverse colon and splenic flexure were treated more quickly than other sites.

In the multivariate analysis, there was variation at patient, practice and health board levels. There was cross-classification between general practice and hospital levels (patients from individual practices were referred to different hospitals) so the hospital level was excluded from the model (hospital type was included at the health board level). Odds of treatment within 90 days were higher for those with palpable masses, abdominal pain or tenesmus, and who consulted more frequently (Table 5

**Table 5:** Results of multilevel modelling of subject level variables on treatment for colorectal cancer within 90 days (yes/no) with levels subject, practice and health board

	**Odds ratio (95% confidence intervals)^a^ *N*=1223**
*Individual variables*	
Age (+1 year)	1.3 (1.12–1.51)
Age squared (+1)	0.998 (0.996–0.999)
Gender (male *vs* female)	1.36 (0.98–1.89)
Palpable rectal mass (yes *vs* no)	8.94 (2.48–32.13)
Palpable abdominal mass (yes *vs* no)	4.32 (1.95–9.59)
Tenesmus (yes *vs* no)	2.69 (1.02–7.09)
Abdominal pain (yes *vs* no)	1.51 (1.06–2.14)
Frequency of consultations (+1 category^b^)	3.25 (2.75–3.85)
History of anxiety/depression (yes *vs* no)	0.47 (0.28–0.77)
On iron at presentation (yes *vs* no)	3.77 (1.40–10.12)
Tumour site	
Right side colon to hepatic flexure	1
Transverse colon to splenic flexure	2.27 (1.25–4.12)
Descending and sigmoid colon	1.01 (0.63–1.60)
Rectum and recto-sigmoid	1.40 (0.90–2.18)
Unspecified or overlapping	1.26 (0.58–2.74)
	
*Practice level variables*	
Number of female GPs (+1)	1.19 (0.99–1.44)
	
	*Variation (standard error) at*
	Health board level 0.045 (0.055)
	Practice level 0.048 (0.112)
	No significant extra-binomial variation
	No significant effect of hospital type at health board level

a Odds ratio higher than unity means higher odds of being treated within 90 days. For interpretation of odds ratios see table 3, footnote 1.

b Frequency of consultation categories: <0.5 consultations per month (from presentation to referral), 0.5–2.5 consultations per month, 2.5–10 consultations per month, >10 consultations per month.

). Age remained significant, following a quadratic relationship, meaning treatment was slower for the old and young. People with a history of anxiety or depression were only half as likely to be treated within 90 days and those on iron therapy at presentation were more likely to be treated quickly. Treatment appeared to be slower for women and faster for patients registered at practices with higher numbers of female GPs, although these differences were not statistically significant.

## DISCUSSION

We found no evidence that people living further from cancer centres received slower treatment. For breast cancer they were, by being referred initially to local general or community hospitals, treated more quickly. Speed of treatment appears to be largely determined by features of the tumour and the symptoms and signs it generates, but age is important in both cancers, type of hospital of referral in breast cancer and general practice factors in colorectal cancer.

The study benefited from a high rate of case note retrieval and information from primary care, where the majority of cancers present. Case notes in primary care have the advantage of being contemporaneous records, but also have some limitations. It is possible that some symptoms were not recorded at time of presentation, but our analysis is likely to include the dominant symptoms and reasons why people consulted. We set strict rules and protocols for data collection to ensure consistency but must accept that, for colorectal cancer, it may be difficult to define the point at which cancer symptoms present in patients with pre-existing bowel disease. That we did not, after adjustments, find delays in treatment for this group suggests that any effect from this, if it occurred, was not substantial. Prospectively, we identified and collected data on as many variables as possible thought to affect delay. This had the advantage of ensuring that possible associations with geographical and organisational factors were not biased by caseload – and indeed, after adjusting for clinical factors, most of these associations disappeared – but the disadvantage is the possibility of some associations emerging by chance. While, then, we can have confidence in our findings regarding urban rural differences (our *a priori* hypothesis), our findings on other factors would benefit from confirmation in other research.

Our findings demonstrate that clinical factors at presentation – symptoms, signs, and tumour properties – are the most important factors in determining time taken before treatment. For breast cancer, our best fitting model for rapid treatment contained the usual sign of breast cancer (a lump), one of locally advanced disease (change in skin contour) and another of regional spread (axillary or supraclavicular lymphadenopathy). The time taken to treat colorectal cancer was usually considerably longer than that for breast cancer – average times to treatment of nearly 5 months are worrying and could plausibly affect survival (Richards *et al*, 1999). Delays were associated particularly with the presence or absence of certain symptoms (e.g. a palpable mass, abdominal pain or tenesmus) and frequency of consultation. Tumours of the transverse colon and splenic flexure were treated more quickly, perhaps because of a high proportion of splenic flexure tumours presenting with obstruction (Mulcahy and O'Donoghue, 1997).

Several other factors we found to be related to delay may be concerned with medical perception of risk. Older people, whose risk of cancer is greater, were treated more quickly than the under 50s, whose symptoms are less likely to represent cancer (Selvachandran *et al*, 2002). In colorectal cancer, the odds of delayed diagnosis were doubled by a past history of anxiety and depression, perhaps because benign causes of gastrointestinal symptoms are thought common in this group so, when presenting with nonspecific symptoms, their risk of cancer is perceived to be low. Men with colorectal cancer were treated more quickly than women, but this difference appears to be largely explained by other factors because it was no longer significant in the multivariate model. Faster treatment for those on iron at presentation is odd. Given our protocols for data collection, this group of patients had not been anaemic for 2 years, so are likely to have a longstanding history of iron deficiency. Perhaps, their risk of colorectal cancer with a given presentation was perceived to be greater – or perhaps this was a chance finding.

Although average times to treatment of colorectal cancer were much longer than breast, they were similar when both presented with a palpable mass. This suggests that much of the delay in colorectal cancer is related to the nonspecific nature of its early symptoms and signs (Selvachandran *et al*, 2002; Tan *et al*, 2002). First, there may be reluctance to expose these patients to investigations that carry a small but significant risk (SIGN, 2003). Second, limited investigative resources in our health care system mean that rapid investigation and treatment are often only available for selected patients – deemed ‘urgent’ – and selection is clearly difficult. In line with this, treatment was faster in the private sector, where investigations are more readily available – although the average time saving compared with cancer centres or general hospitals was only about 3 weeks.

Adjusting for the above factors, we found few associations with geographical, socioeconomic or organisational variables. At the population level, neither rural residence nor socioeconomic deprivation were associated with delays in treatment, so provider delays do not explain the higher chance of advanced disease reported for these groups of patients (Macleod *et al*, 2000; Campbell *et al*, 2001). Alternative explanations should be sought, including the time taken by patients before presentation (Bain and Campbell, 2000).

Three organisational factors appeared, however, to be important. First, breast cancer was treated most quickly if detected by screening. This appears to show the benefits of a well-organised, systematic approach to detection and treatment. More than 80% of people with breast cancer presented outside the screening programme in our study, and this group might benefit from a similarly efficient management pathway. Second, adjusting for clinical factors, treatment delays were more likely for women with breast cancer referred to cancer centres than other hospitals. This problem with centralised care has been reported for other cancers (Milne *et al*, 2000), but must be balanced against the possible benefits of specialist treatment (Mikeljevic *et al*, 2003). Finally, time to treatment of colorectal cancer varied among general practices, but this variation was not adequately explained by the factors we considered. There was a suggestion that treatment may be faster in practices with more female general practitioners, but no evidence of systematic differences between practices of different size or for those with longer waiting times for appointments.

In conclusion, the time taken to treat breast and colorectal cancers appears to be due largely to characteristics of the tumour and how it presents. Long delays are common for colorectal cancer, but we found that time to treatment was no greater for people with many factors previously suspected to cause delay – including geographical factors, deprivation and many general practice factors.
